# Decreased Expression of the EAAT5 Glutamate Transporter at Photoreceptor Synapses in Early, Pre-Clinical Experimental Autoimmune Encephalomyelitis, a Mouse Model of Multiple Sclerosis

**DOI:** 10.3390/biomedicines12112545

**Published:** 2024-11-07

**Authors:** Ali El Samad, Julia Jaffal, Dalia R. Ibrahim, Karin Schwarz, Frank Schmitz

**Affiliations:** Institute of Anatomy, Department of Neuroanatomy, Medical School Homburg, Saarland University, 66421 Homburg, Germany; ali.elsamad.94@hotmail.com (A.E.S.); juliajaffal8@gmail.com (J.J.); daliaibrahim93@outlook.com (D.R.I.); karin.schwarz@uks.eu (K.S.)

**Keywords:** multiple sclerosis, EAE, retina, photoreceptor synapse, EAAT5 (SLC1A7), glutamate transporter

## Abstract

Background: Multiple sclerosis is a frequent neuroinflammatory and neurodegenerative disease of the central nervous system that includes alterations in the white and gray matter of the brain. The visual system is frequently affected in multiple sclerosis. Glutamate excitotoxicity might play a role in disease pathogenesis. Methodology: In the present study, we analyzed with qualitative and quantitative immunofluorescence microscopy and Western blot analyses whether alterations in the EAAT5 (SLC1A7) glutamate transporter could be involved in the previously observed alterations in structure and function of glutamatergic photoreceptor ribbon synapses in the EAE mouse model of MS. EAAT5 is a presynaptic glutamate transporter located near the presynaptic release sites. Results: We found that EAAT5 was strongly reduced at the photoreceptor synapses of EAE retinas in comparison to the photoreceptor synapses of the respective control retinas as early as day 9 post-immunization. The Western blot analyses demonstrated a decreased EAAT5 expression in EAE retinas. Conclusions: Our data illustrate early alterations of the EAAT5 glutamate transporter in the early pre-clinical phase of EAE/MS and suggest an involvement of EAAT5 in the previously observed early synaptic changes at photoreceptor synapses. The precise mechanisms need to be elucidated by future investigations.

## 1. Introduction

Multiple sclerosis (MS) is a chronic autoimmune disease of the central nervous system (CNS). Neuroinflammation and neurodegeneration play an important role in MS. The disease results in axonal fiber tract damage/demyelination in the white matter [1,2,3,4]. Alterations in MS are not restricted to the white matter of the CNS but are present also in the gray matter of the CNS (e.g., [5,6,7,8,9]). Gray matter alterations include abnormalities of synapses and synapse networks as well as neurodegeneration and were observed in different brain regions of MS patients and in animal models of MS (for review, [10,11,12,13]).

It is well known that glutamate levels are elevated in MS, e.g., in the cerebrospinal fluid of MS patients, suggesting that glutamate excitotoxicity and possibly dysfunctions of glutamatergic synaptic signaling might play a role in the pathogenesis of MS ([14,15,16,17,18]; for review, see [12,19,20,21,22,23]). Multiple sources from neurons, glial- and immune cells could contribute to elevated extracellular glutamate levels [12,22,23,24].

The visual system is frequently affected in MS and inflammation of the optic nerve (optic neuritis) is an early symptom in MS. In animal models of MS, retinal changes included degeneration of retinal ganglion cells and alterations in synapse structure and function that could be inflammation-driven (e.g., [12,25,26,27,28]). Changes in photoreceptor synapses occurred early in the pre-clinical phase of EAE before obvious alterations in the optic nerve [25,26,27]. The molecular mechanisms of these early synaptic changes are not completely understood.

In previous studies ([25,26,27]; for review, [13]), we observed alterations of photoreceptor synapses in the EAE mouse model of MS on day 9 after injection. Photoreceptor synapses are continuously active glutamatergic ribbon synapses that contain presynaptic ribbons as eponymous structural specialization [29]. Synaptic ribbons are anchored to the presynaptic release sites and bind large numbers of glutamatergic synaptic vesicles to promote continuous synaptic vesicle exocytosis. RIBEYE is a main and unique component of synaptic ribbons [30,31,32]. In general, glutamate transporters remove glutamate released by synaptic transmission from the synaptic cleft and transport glutamate either into neuronal cells or glial cells to prevent glutamatergic excitotoxicity [12,33,34].

In the present study, we investigated whether an imbalance of glutamate homeostasis caused by alterations of glutamate transporters could possibly contribute to photoreceptor synapse pathology in EAE retinas. Five families of glutamate transporters have been cloned and functionally characterized [33,34,35,36,37,38,39,40]. In our analyses, we focused on the EAAT5 (SLC1A7) glutamate plasma membrane transporter that is strongly expressed in the retina [40,41,42,43,44,45,46]. EAAT5 is known to be localized in close vicinity of the presynaptic release sites of ribbon synapses [41,42,43,44,45,46]. We analyzed EAAT5 expression in the retina of MOG/CFA-injected EAE mice (9 days after injection) in comparison to control-injected mice. We found that EAAT5 expression at the presynaptic release site of photoreceptor synapses is strongly decreased in EAE mice in comparison to control mice, suggesting that malfunctions of glutamate transporters/glutamate clearance could contribute to the previously observed synapse pathology in EAE.

## 2. Materials and Methods

### 2.1. Animals

All procedures concerning laboratory animals were reviewed and approved by the local animal authorities (Tierschutzbeauftragte der Universität des Saarlandes and Landesamt für Verbraucherschutz; Geschäftsbereich 3; 66115 Saarbrücken, Germany; GB 3-2.4.2.2-25-2020). Female C57BL/6J mice older than 10 weeks and with a body weight between 20 g and 25 g were used for EAE induction, as previously described [25,26,27,47]. Mice were kept on a 10 h light–14 h dark cycle and provided with standard food and water ad libitum.

### 2.2. Antibodies (Table 1 and Table 2)

### 2.3. Methods

#### 2.3.1. Induction of EAE in Female Mice

Experimental autoimmune encephalomyelitis (EAE) was induced by immunizing 10–12 week old female C57BL/6J mice (20–25 g body weight) with MOG_35–55_ peptide of mouse myelin oligodendrocyte glycoprotein as previously described [25,26,27,51]. MS is a disease that predominantly affects young female adults in humans. For the EAE model, we also used only female mice, as most of the EAE studies do (e.g., [25,26,27,52,53,54]). Mice were either injected with a ready-to-go emulsion from Hooke Laboratories (MOG_35–55_/CFA Emulsion PTX, Hooke Laboratories, Lawrence, MA, USA; #EK-2110; 1 mg MOG peptide/mL of emulsion) or with lab-made emulsions. For the preparation of lab-made MOG/CFA suspensions, MOG_35–55_ peptide was dissolved in sterile water (2 mg/mL) and emulsified in a one-to-one ratio with complete Freund adjuvant (CFA), that is composed of incomplete Freund adjuvant (iCFA, Sigma; #F5506) to which 10 mg/mL inactivated *Mycobacterium tuberculosis* were added (Fisher Scientific; Schwerte, Germany; 10218823). For control emulsion (CFA), complete Freund adjuvant was emulsified in a one-to-one ratio with sterile water. A total of 200 μL of the respective emulsion (either MOG/CFA (experimental group) or CFA (control group)) was subcutaneously administered in the axillary and groin region of the mice. Blood–brain barrier permeability was enhanced by two intraperitoneal injections of 200 ng pertussis toxin (PTX) from *B. pertussis* (List Biological Laboratories, Campbell, CA, USA; #181). The first one on the day of immunization (60 min after application of emulsion) and the second one on the subsequent day (16–20 h after immunization). Five independent immunizations, each composed of CFA-injected control animals and MOG/CFA-injected experimental animals, were performed for immunofluorescence microscopy and 5 independent immunizations, each composed of CFA-injected control animals and MOG/CFA-injected experimental animals, were performed for Western blot analyses. In these injections, animals were randomly allocated to the respective groups (i.e., control group or experimental group) and housed in the same cage.

#### 2.3.2. Cloning of pET28a-EAAT5

In brief, cDNA encoding amino acids 100–250 of human EAAT5 were cloned into the vector pET-28a via Gibson assembly [55]. For this purpose, pET-28a vector was linearized by restriction digest with *NheI* and *XhoI*. The insert was provided as a synthetic DNA construct (gBlock, Integrated DNA Technologies (IDT), Coralville, IA, USA). The gBlock contained 34 nucleotides of overlapping vector sequences at both ends. At 5′ end of the gBlock vector sequence was followed by a *NheI* restriction site, a STREP-Tag II and the sequence encoding amino acids 100-250 of human EAAT5. The 3′ end of the gBlock was completed with 34 nucleotides of vector sequence, including the *XhoI* restriction site. In the resulting recombinant vector, EAAT5 is expressed as fusion protein containing a N-terminal hexa-His-Tag, followed by a thrombin cutting side and the STREP-Tag II. At the C-terminal end, the resulting fusion protein contains a second hexa-His-tag. Gibson assembly was performed according to the manufacturer’s protocol using the Gibson assembly cloning kit (New England Biolabs, Frankfurt am Main, Germany).

#### 2.3.3. Cloning of pET28a-Cre (Control Protein)

Fusion protein comprising the membrane permeable HIV TAT peptide followed in frame by Cre recombinase was cloned into the vector pET-28a via Gibson assembly [55]. For this, the vector was linearized by restriction digest using *NheI* and *XhoI*. The insert was provided as synthetic DNA construct (gBlock, Integrated DNA Technologies (IDT), Coralville, IA, USA). The gBlock contained 34 nucleotides of overlapping vector sequences at both ends. The 5′ end of the gBlock vector sequence was followed by a *NheI* restriction site, a STREP-Tag II, and the sequence encoding the TAT-Cre fusion protein. The 3′ end of the gBlock was completed by 34 nucleotides of vector sequence. In the resulting cloned vector, TAT-Cre is expressed as fusion protein containing a N-terminal hexa-His-Tag, followed by a thrombin cutting side and the STREP-Tag II. Gibson assembly was performed according to the manufacturer’s protocol using the Gibson assembly cloning kit (New England Biolabs; Frankfurt am Main, Germany).

#### 2.3.4. Fusion Protein Expression and Purification

Fusion protein expression was conducted in BL21 T7 Express bacteria. Transformed bacteria were grown in LB medium supplemented with 2% glucose and kanamycin (final concentration 10 µg/mL) at 37 °C until they reached OD_600_ = 0.8. Expression of fusion protein was induced by adding IPTG to a final concentration of 0.1 mM. After 5 h of induction with IPTG (at RT), bacteria were harvested by centrifugation. The bacterial pellet was washed several times with ice cold PBS and was finally resuspended in imidazole lysis buffer (300 mM NaCl, 50 mM NaH_2_PO_4_, 2.5 mM imidazole, pH 8.0) supplemented with lysozyme (1 mg/mL). After 30 min incubation of ice, followed by sonication, the bacterial lysate was cleared by centrifugation (10,000× *g*; 30 min 4 °C). Cleared lysate was incubated with Ni-NTA agarose overnight on an overhead rotator at 4 °C to allow binding of fusion protein to the Ni-NTA matrix (1 mL of Ni-NTA matrix/500 mL of bacterial culture). Next, lysate-Ni-NTA mixture was loaded into a column and flow thru was collected for SDS-Page analysis. The column was than washed with 5 bed volumes of washing buffers containing increasing concentrations of imidazole, starting with 10 mM imidazole (300 mM NaCl, 50 mM NaH_2_PO_4_, 10 mM imidazole, pH 8.0), followed by washes with 20 mM imidazole (300 mM NaCl, 50 mM NaH_2_PO_4_, 20 mM imidazole, pH 8.0) and 250 mM imidazole (300 mM NaCl, 50 mM NaH_2_PO_4_, 250 mM imidazole, pH 8.0). To elute the bound fusion protein, 6 mL of elution buffer (300 mM NaCl, 50 mM NaH_2_PO_4_, 400 mM imidazole, pH 8.0) were applied to the column and flow thru was collected in 0.5 mL fractions. EAAT5 fusion protein was enriched in faction 8–10 as assessed by SDS-PAGE and Western blotting.

#### 2.3.5. Pre-Absorption of EAAT5 Antibody for Immunolabeling Experiments

Pre-absorption blocking experiments were performed to verify the specificity of EAAT5 antibody (Abcam, ab230217, Table 1). First, we determined the suitable working dilution (1:200, Table 1) of the EAAT5 antibody, which results in an antibody protein concentration of ~167 nM. The corresponding EAAT5 blocking fusion protein against which EAAT5 antibody was raised, as well as an unrelated fusion protein (HexaHIS-tagged Cre recombinase) were mixed with the EAAT5 antibody in a molar ratio of 5:1 in different tubes (an experimental tube and control tube). Both tubes were incubated on a rotator overnight at 4 °C. On the following day, both tubes were centrifuged at 30,000 rpm (Biofuge Stratos centrifuge, #3331 rotor; ThermoFisher; Waltham, MA, USA) for 5 min. The supernatants were employed for immunostaining experiments. Different semi-thin sections were co-immunolabeled simultaneously. One was incubated with mouse monoclonal RIBEYE antibody 2D9 and the EAAT5 antibody that was pre-absorbed with EAAT5 fusion protein as described above. In parallel, another section was double immunostained with EAAT5 antibody pre-absorbed with the unrelated fusion protein (HexaHIS-tagged Cre) along with anti-RIBEYE antibody 2D9. The antibodies against RIBEYE served as reference immunosignals. Both incubations were performed overnight at 4 °C. On the next day, sections were washed multiple times with PBS to remove unbound primary antibodies and were subsequently incubated with the corresponding fluorescent conjugated secondary antibody for 2 h at RT (see Table 2). Binding of the EAAT5 rabbit polyclonal antibody was detected with donkey anti-rabbit immunoglobulins conjugated to Alexa 488 and binding of the mouse monoclonal RIBEYE antibody (clone 2D9) was detected with donkey anti-mouse immunoglobulins conjugated to Alexa 568. Lastly, the sections were washed five times (5 min each) with PBS and mounted in n-propyl gallate (NPG) antifade solution, as previously described [25,30,56,57,58,59]. Negative control incubations were performed under the same conditions as described above, but in the absence of the primary antibody to check for non-specific fluorescence signals, e.g., by autofluorescence.

#### 2.3.6. Immunolabeling of Retinal Sections

Immunofluorescence microscopy was performed on semi-thin retinal resin sections from MOG/CFA-injected EAE mice and CFA-injected control mice, as previously described [25,26,27]. For this purpose, eyes were isolated from the respective mice within 5 min post-mortem. The anterior eyecup was removed as described [25,26,27,60]. The posterior eyecup with the attached retina was flash-frozen in liquid-nitrogen-cooled isopentane and freeze-dried in a vacuum generated by a DUO 004B vacuum pump (Arthur-Pfeiffer Vakuumtechnik, Wetzlar, Germany), as previously described [25,26,27,48,56,57,60]. During lyophilization, the samples were cooled with liquid nitrogen for ~two days. After that, the samples were equilibrated to room temperature and infiltrated for ~48 h with Epon resin, as described [25,26,27,60]. Infiltration with Epon resin was performed the first 12 h at 28 °C in an overhead rotator at 2rpm to ease infiltration of the samples with Epon. Afterwards, the infiltration with Epon resin was continued at RT. After infiltration with Epon resin, samples were polymerized for 2 days at 60 °C. From the hardened tissue blocks, 0.5 µm thin (semi-thin) sections were cut with a Reichert ultramicrotome using a diamond knife (Diatome; Nidau, Switzerland) to generate standardized sections of identical thickness. Sections were collected on glass coverslips. The Epon resin was removed from the semi-thin sections and processed for immunocytochemistry, as previously described [25,26,27,48,56,57,60]. The sections were processed for double-immunolabeling with the indicated antibodies and incubated overnight at 4 °C in the indicated optimized primary antibody dilutions (see Table 1). In each experiment, a reference (CFA) and experimental samples (MOG/CFA) were processed simultaneously under identical conditions. The next day, sections were washed several times with PBS to remove unbound primary antibodies and subsequently incubated with the corresponding fluorophore-conjugated secondary antibodies (Table 2) for 1 h at room temperature (RT). Finally, sections were washed again several times with PBS and were embedded in NPG-antifade, as described [25,48,56,57,58,61].

#### 2.3.7. Confocal Microscopy and Quantitative Analyses of Immunosignals

Confocal microscopy was performed with a Nikon A1R confocal microscope (Düsseldorf, Germany), as previously described [25,56,57,58,59]. Images were acquired with a 60×/1.40 N.A. oil objective under the control of NIS Elements software (NIS Elements AR 3.2, 64 bit; Düsseldorf, Germany). In the individual experiments, image acquisition from MOG/CFA- and CFA-samples was performed under identical conditions by using the re-use image settings option in the NIS Elements software. Image acquisition and analyses were performed in a blinded manner with the experimenter not knowing whether the samples were from CFA- or MOG/CFA-injected mice. CFA- and MOG/CFA-injected samples from each embedding were imaged under identical conditions using the re-use option of the NIS Elements software. Five independent experiments were performed for each experimental group. For quantification, an identical rectangular ROI was used for both samples and placed along the OPL that could be unambiguously identified by the actin and EAAT5 immunosignals. ROIs were managed with the Analyze-Tools-ROI Manager of ImageJ. EAAT5 and actin immunosignals were simultaneously recorded. Actin signals served as a reference signal to correct for potential differences in section thickness. Actin is particularly suitable as a reference protein because actin turned out to remain unchanged between CFA- and MOG/CFA-injected samples [25]. Fluorescence intensities of both proteins were measured as integrated density. EAAT5 immunosignal integrated densities were normalized to the corresponding actin integrated density values. The arithmetic mean values of CFA were set to 100% and the MOG/CFA values were related to them. Integrated density values were analyzed with Microsoft Excel. Statistical analyses were performed with GraphPad Prism 10 (version 10.2.3), see below.

#### 2.3.8. Statistical Analyses of Immunofluorescence Signals

Statistical analyses were performed with GraphPad 10 (version 10.2.3). Based on our experience and previously performed a priori sample size estimations (α ≤ 0.05; effect size Cohen’s d = 0.8; power = 0.8) using G*Power Version 3.1.9.6 [62], a total of 5 independent immunizations, each composed of CFA-injected control animals and MOG/CFA-injected experimental animals, were performed. In these injections, animals were randomly allocated to the respective groups (i.e., control group or experimental group) and housed in the same cage. For quantification, samples were randomly drawn from these five independent injections and analyzed by immunofluorescence. First, we tested whether data from these individual experiments could be pooled. For this purpose, we determined whether the reference/control group (CFA) from the independent individual experiments differed significantly from each other. To decide this, we determined whether data were normally distributed using the Shapiro–Wilk test. Not all data were normally distributed. Next, we used Kruskal–Wallis ANOVA and post hoc Dunn tests to compare the different CFA reference groups from the individual experiments. The values of the individual CFA reference (control) animals did not differ significantly from each other in the different independent experiments. Therefore, we pooled the data for further analysis. Pooled data from CFA and MOG/CFA samples were analyzed for statistical differences by the non-parametric Mann–Whitney *U* test. Differences were considered statistically different with *p* < 0.05. Post hoc analysis of data obtained for immunofluorescence data showed a power of 0.985 with an effect size Hedges’ g = 0.953835 and α = 0.0001 [63].

#### 2.3.9. Miscellaneous Procedures

##### Western Blot (WB) Analyses

The specificity of EAAT5 antibody was analyzed by Western blot (WB) with retinal lysates from wild-type mice. Retinal lysate was prepared as described next. In order to analyze possible changes in the global expression of EAAT5 in EAE retinas in comparison to control retinas, retina extracts from MOG/CFA-injected EAE mice and CFA-injected control mice were prepared in ice-cold RIPA lysis buffer containing 150 mM NaCl, 50 mM Tris, pH 7.4, 1% NP-40, 0.5% sodium deoxycholate, 0.1% SDS, and cOMPLETE EDTA-free protease inhibitor cocktail (Roche; COEDTAF-RO). An Ultra Turrax T8A (IKA Labortechnik; Staufen im Breisgau, Germany) was used to homogenize the retinas for 1–2 s. After this step, the retinal lysate was kept in the RIPA lysis buffer for 20 min on ice (with gentle agitation) and then centrifuged at 13,000 rpm for 30 min (at 4 °C). The pellet was discarded while the supernatant was collected in a new tube, mixed 1:1 (*v*/*v*) with SDS Laemmli buffer and heated for 10 min to 96 °C. Protein quantification was performed with the Amidoblack method [64]. For this purpose, 5 μL of protein sample dissolved in SDS-Laemmli buffer and BSA standards were spotted on cellulose acetate membranes, air dried, and stained with an Amido Black 10B solution (0.5% (*w*/*v*) Amido Black 10B in methanol (45%, *v*/*v*)/water (45% *v*/*v*)/10% acetic acid (10%, *v*/*v*)) for 10 min at RT. Next, the cellulose acetate sheets were washed three times with 1 mL wash solution (47.5% each of methanol and water, and 5% glacial acetic acid) (5 min each). The stained cellulose acetate sheets were then dried again at RT and the individual samples were cut apart. The individual samples were transferred into separate test tubes (2 mL Eppendorf cups) and dissolved in 1 mL dissolution solution (80% (*v*/*v*) formic acid, 10% (*v*/*v*) acetic acid and 10% (*w*/*v*) trichloro acetic acid). For complete dissolution, the membrane pieces were incubated at 50 °C under constant shaking for 30 min followed by measuring the absorbance of the resulting solution at 620 nm using a UV/Visible spectrophotometer (Pharmacia-Pfizer, New York, NY, USA). Protein concentrations were determined by comparing the absorbance values to a BSA standard curve. We applied 30 μg of protein of the retinal lysates on each lane and separated by 10% acrylamide SDS-PAGE. After SDS-PAGE, proteins were electro-transferred from the resolving gel to nitrocellulose membrane at 50 volts for 6.5 h at 4 °C. On the next day, the membrane with the electro-transferred proteins was washed several times with PBS and incubated with 5% (*w*/*v*) non-fatty dry milk in PBS for 1 hr at room temperature (RT) to block non-specific binding sites and incubated with the primary antibody dilutions indicated in Table 1 (on a shaker, overnight at 4 °C). After this, the membrane was washed several times with PBS to remove unbound primary antibody and incubated in the corresponding secondary antibody conjugated with horseradish peroxidase (HRP) with the antibody dilutions as given in Table 2 with gentle shaking (for 2 h at RT). Later, membranes were washed three times (10 min each) to remove unbound secondary antibody. Antibody binding was visualized by chemiluminescence (ECL) acquired by Bio-Rad Gel Doc imaging systems (Bio-Rad ChemiDoc^TM^ MP Imaging System and Bio-Rad ChemiDoc^TM^ XRS Imaging System; Feldkirchen, Germany). For re-probing of WB membranes, membranes were incubated with 0.2 M glycine and 0.1% SDS, pH 2.2, in H_2_O (stripping buffer) for 20 min at room temperature with mild shaking to remove bound antibodies. Then, the membrane was washed 3 times (10 min each) with TBST (Tris-buffered saline with 0.1% (*w*/*v*) Tween^®^ 20 detergent) to remove the stripping buffer. Afterwards, the membrane was incubated with 5% (*w*/*v*) non-fatty dry milk in PBS for 1 h (RT) to saturate non-specific protein binding sites. After that, the membrane was probed with the mouse monoclonal anti-actin primary antibody (clone C4 at the dilution given in Table 1). Binding of mouse monoclonal actin antibody was detected with the corresponding secondary antibody (Table 2) and visualized via ECL, as described above.

##### Quantification of WB Bands and Statistical Analyses

Image Studio Lite software (Li-Cor, version 5.2) was employed to analyze the band chemoluminescence signal corresponding to the global expression of EAAT5 protein present in whole retinal lysate samples from independent experiments. Five independent experiments were analyzed on pairs of retinal lysates from CFA and MOG/CFA mice. Using the analysis option of the Image Studio Lite software (Li-Cor, version 5.2), a rectangular ROI was drawn specifically around the targeted band to measure the chemoluminescence pixel intensity sum corrected for area and background (signal value). The band intensity of the EAAT5 target protein was normalized to the reference protein (actin) band intensity in the same lane to correct for possible loading differences. Actin was shown to remain unchanged between MOG/CFA- and CFA-injected samples [25]. For statistical analysis, the normalized EAAT5 band intensity values of each CFA sample were set to 100% and the corresponding MOG/CFA normalized EAAT5 band intensity value was related to it. Next, these normalized data were exported and statistically analyzed using GraphPad prism 10. MOG/CFA values were normally distributed according to Shapiro–Wilk tests. Therefore, one sample *t*-test was used for statistical difference analysis and *p* < 0.05 was considered a significant difference. Values were calculated and plotted as arithmetic means and standard errors of the mean (S.E.M.) with GraphPad prism 10. All individual values were depicted.

## 3. Results

In the present study, we analyzed the expression of the glutamate transporter EAAT5 in photoreceptor synapses of the outer plexiform layer (OPL) in the mouse retina by immunofluorescence microscopy. We compared retinas from MOG/CFA-injected EAE mice with retinas from CFA-injected control mice on day 9 after injection. For immunocytochemistry, we used an antigen affinity-purified polyclonal antibody against EAAT5.

**Figure 1:**
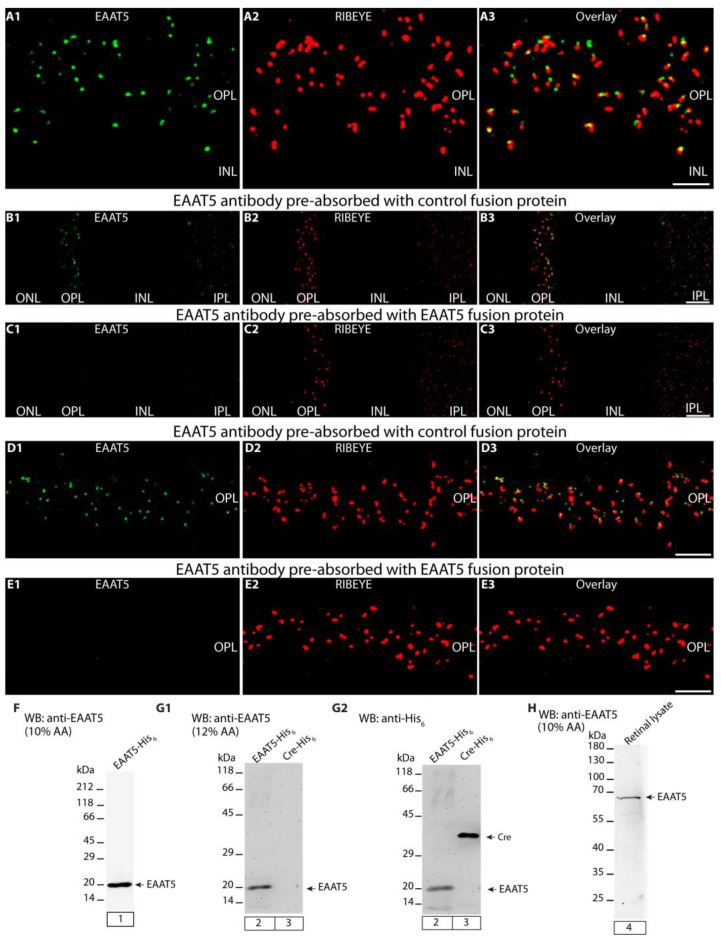
Validation of the specificity of antigen affinity-purified rabbit polyclonal EAAT5 antibody. (**A1**,**A2**) A magnified micrograph of the OPL double-immunolabeled with affinity-purified rabbit polyclonal anti-EAAT5 antibody in green and with the mouse monoclonal anti-RIBEYE (2D9) antibody in red, respectively. (**A3**) A merged image for both antibodies in the OPL of photoreceptor synapses. (**B1**–**E3**) The pre-absorption experiments of EAAT5 signals performed on 0.5 μm-thin (semi-thin) wild-type retina sections. (**B1**,**D1**) The EAAT5 antibody was pre-absorbed with either an unrelated control fusion protein or (**C1**,**E1**) with the EAAT5 fusion protein against which the antibody was raised. (**B2**,**C2**,**D2**,**E2**) The RIBEYE immunosignals were unaffected by blocking with either fusion protein. (**B3**,**C3**,**D3**,**E3**) The signals from the respective red and green channels were overlaid. (**F**–**H**) Western blots (WB) analyses of the EAAT5 antibody that was generated against EAAT5 fusion protein. (**F**,**G1**) The EAAT5 antibody detects a ~20 kDa band in lane 1 and 2 corresponding to the EAAT5 fusion protein. (**G1**) Lane 3 containing Cre control fusion protein did not show a band/reactivity with the EAAT5 antibody thus demonstrating the specificity of the antigen affinity-purified polyclonal EAAT5 antibody. (**G2**) shows anti-HexaHIS antibody incubation of the very same blot strip in G1, manifesting the loading of both EAAT5 and Cre control fusion protein. (H) single band ≈ 65 kDa is detected in retina lysate from wild-type mice by EAAT5 antibody. Abbreviations: OPL, outer plexiform layer. Scale bars: 5 µm.

The EAAT5 antibody produced strong punctate signals in the OPL near the presynaptic release sites of photoreceptor synapses (Figure 1A1–A3). The presynaptic release sites were immunolabeled with mouse monoclonal antibodies against RIBEYE (clone 2D9) (Figure 1A2,A3). RIBEYE is the main component of synaptic ribbons [30,31,32]. This punctate EAAT5 immunosignal near the presynaptic release sites was expected because a very similar immunolabeling pattern was previously observed for EAAT5 in the mouse retina [46].

In addition to a strong EAAT5 immunosignal in the OPL, we also observed a punctate EAAT5 immunolabeling pattern in the inner plexiform layer (IPL) (Figure 1B1,B3) that represents EAAT5 in the presynaptic terminals of rod bipolar cells, as previously reported by another group [46].

The EAAT5 immunosignals in the OPL and IPL were completely abolished if the EAAT5 antibody was pre-absorbed with the EAAT5 fusion protein against which it was raised (Figure 1C1,C3,E1,E3). The RIBEYE immunosignals were un-affected by the pre-absorption of the EAAT5 antibody with the EAAT5 fusion protein (Figure 1C2,E2). The EAAT5 immunosignals (as well as the RIBEYE immunosignals) remained unaffected if the EAAT5 antibody was pre-absorbed with an irrelevant HIS-tagged fusion protein (Figure 1B1–B3,D1–D3) demonstrating the specificity of the immunolabeling data in the pre-absorption experiment.

In Western blot analyses, the EAAT5 antibody detected the recombinant, bacterially expressed EAAT5 fusion protein it was raised against (Figure 1F, lane 1; Figure 1G1, lane 2) but not the Cre control fusion protein (Figure 1G1, lane 2), further indicating the specificity of the antibody. In Figure 1G2, both fusion proteins from the same blot as shown in Figure 1G1 were incubated with anti-HexaHIS monoclonal antibody to visualize both proteins and to document the loading of the EAAT5 fusion protein and Cre control protein on the WB membrane. The EAAT5 antibody detected a protein band at the expected running position of ≈65 kDa in wild-type mouse retinal lysates (Figure 1H).

**Figure 2:**
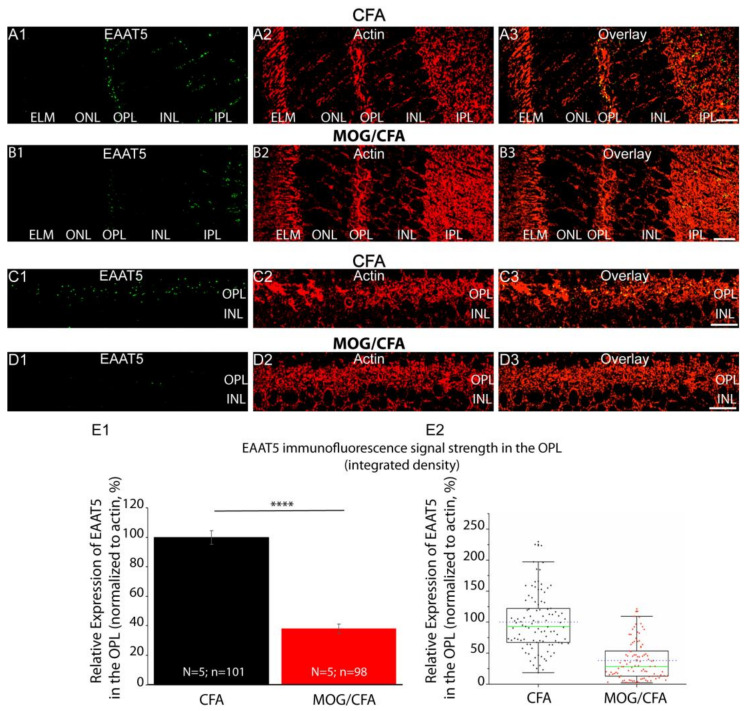
EAAT5 immunofluorescence signals are strongly reduced in photoreceptor synapses in OPL from MOG/CFA-injected EAE mice in comparison to CFA-injected control mice. (**A1**–**D3**) Double immunolabeling of 0.5 µm-thin (semi-thin) retina sections from CFA-injected control mice and MOG/CFA-injected EAE mice (day 9 post injection) with mouse monoclonal antibody against actin (clone C4, in red channel) and rabbit polyclonal antibody against EAAT5 (in green channel). Immunosignals from respective green and red channels were superimposed in (**A3**,**B3**,**C3**,**D3**). (**C1**–**D3**) Zoomed view of OPL that is double immuno-labeled with EAAT5 and RIBEYE antibodies. (**E1**) Histogram depicts mean fluorescence intensities (%) of EAAT5 immunosignals of controls (CFA) and EAE mice (MOG/CFA) in OPL. Values in (**E1**) are means ± S.E.M. (****, *p* ≤ 0.0001). (**E2**) Box and whisker diagram shows distribution of individual values from (**E1**). Boxes mean and median values denoted by horizontal dashed blue line and solid green line, respectively. Boxes illustrate 25th–75th percentiles of data points, and whiskers represent 1.5 times of interquartile range (IQR). Statistical significance was determined with Mann–Whitney U-test (for details, see Methods Section 2.3). Abbreviations: CFA, complete Freund’s adjuvant; EAE, experimental autoimmune encephalomyelitis; MOG, myelin oligodendrocyte protein; N, number of mice; n, number of confocal images analyzed to quantify integrated densities of fluorescence signals from retinal sections; OPL, outer plexiform layer; S.E.M., standard error of mean. Scale bars: 5 µm.

We used the affinity-purified rabbit polyclonal EAAT5 antibody, that has been validated by the experiments shown in Figure 1, to analyze the expression of EAAT5 in photoreceptor synapses in the OPL from MOG/CFA-injected EAE mice in comparison to photoreceptor synapses in the OPL of CFA-injected littermate control mice by immunohistochemistry (Figure 2). In photoreceptor synapses in the OPL of MOG/CFA-injected EAE mice (Figure 2B1–B3,D1–D3), the EAAT5 immunosignals were strongly reduced in comparison to photoreceptor synapses in the OPL of CFA-injected littermate control mice (Figure 2A1–A3,C1–C3) as evaluated by qualitative (Figure 2A1–A3,B1–B3,C1–C3,D1–D3) and quantitative immunocytochemistry (Figure 2E1,E2). In these immunolabelling analyses, the retinal sections were co-immunolabelled with antibodies against EAAT5 and actin (Figure 2A1–A3,B1–B3,C1–C3,D1–D3). Actin served as reference protein for the visualization of retinal layers (including the OPL) as well as for the quantification/normalization of the EAAT5 immunosignals (Figure 2E1,E2). Actin is a suitable reference protein for this purpose because actin was shown to remain unchanged in EAE at this stage [25].

**Figure 3:**
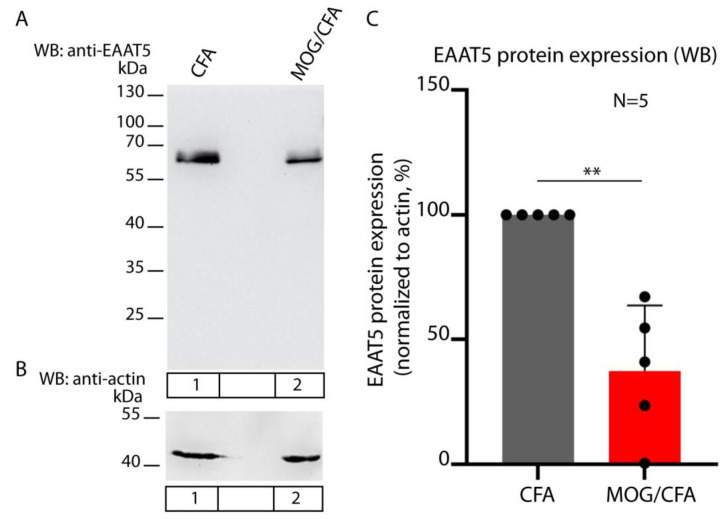
The Western blot (WB) analyses of the total EAAT5 expression in the retinal lysates of MOG/CFA-injected EAE mice and CFA-injected control mice on day 9 after injection. (**A**) The EAAT5 antibody detects EAAT5 at the expected running position of ≈65 kDa in the WB analyses of retinal lysates from CFA-injected control mice and MOG/CFA-injected EAE mice (lanes 1 and 2, respectively). (**B**) shows the same WB membrane as shown in (A) but re-probed with an antibody against actin. Actin (at ≈43 kDa) served as loading control. (**C**) summarizes the results from 5 independent Western blot experiments in which EAAT5 expression was analyzed in retinas from CFA- and MOG/CFA-injected mice (as normalized EAAT5 expression, normalized to the loading control (actin)). CFA control group values were assigned to 100% for better assessment of the relative differences between CFA-injected control mice and MOG/CFA-injected EAE mice. Values in (**C**) are means ± S.E.M. One sample *t*-test was used to determine the statistical significance (*p*-value). Abbreviations: WB, Western blot; CFA, complete Freund’s adjuvant; EAE, experimental autoimmune encephalomyelitis; MOG, myelin oligodendrocyte protein; S.E.M., standard error of the mean; N, number of experiments; **, *p* ≤ 0.01.

The global expression of EAAT5 protein, as judged by the Western blot analyses (Figure 3), was also decreased in the retinas from MOG/CFA-injected EAE mice in comparison to CFA-injected control mice at day 9 after injection.

## 4. Discussion

Multiple sclerosis (MS) is a chronic neuroinflammatory disease of the CNS in which pro-inflammatory cytokines are up-regulated [22]. Pro-inflammatory cytokines were previously reported to cause down-regulation of glutamate transporters in various regions of the CNS (e.g., [45,65,66,67,68,69]; for review, [10,13,70,71]). In some studies, though, an enhanced expression of glutamate transporters was found (e.g., [72]). Of note, a glutamate transporter polymorphism has been observed to be associated with higher glutamate concentrations in relapsing multiple sclerosis, pointing to an important contribution of glutamate transporters in the pathogenesis of MS [73,74].

Glutamate transporters, in general, remove glutamate released by synaptic transmission from the synaptic cleft and physiologically transport glutamate back into neuronal cells or glial cells to prevent glutamatergic excitotoxicity [12,33,34,38,75,76]. Dysfunctional glutamate uptake could lead to glutamate spillover of glutamate from synaptic sites to extrasynaptic sites (for review, [12,75,77]). Binding of glutamate to extrasynaptic glutamate receptors leads to functional impairment and finally cell death via multiple mechanisms and pathways ([78,79,80]; for review, [12,81,82,83]). The mechanisms of glutamatergic excitotoxicity include neurons and glial cells ([80,84]; for review, [12,77,82]).

In the present study, we focused on the EAAT5 glutamate transporter. EAAT5 is located in the presynaptic terminals of retinal neurons, i.e., photoreceptors and bipolar cells, in close proximity to the presynaptic glutamate release sites that are characterized by synaptic ribbons [42,43,44,46].

In the current work, we demonstrated that the presynaptic glutamate transporter EAAT5 is strongly down-regulated in photoreceptor synapses of MOG/CFA-injected EAE mice in comparison to photoreceptor synapses from CFA-injected control mice. Down-regulation of EAAT5 in photoreceptor synapses was demonstrated by qualitative and quantitative immunofluorescence microscopy. In EAE, we observed a strong decrease in EAAT5 at photoreceptor synapses close to the presynaptic ribbons. A decreased expression of EAAT5 was also demonstrated by the Western blot analyses of whole retinal lysates. The physiological consequences of the strongly decreased expression of EAAT5 at photoreceptor ribbon synapses in EAE remain to be elucidated by future investigations. Clearly, the EAAT5 transporter is close to the presynaptic release site and contributes to high temporal resolution of synaptic signaling in the retina as judged by electrophysiological analyses [46]. Interestingly, visual performance/frequence sensitivity is also compromised in EAE as measured by optometry [25]. The decreased expression of EAAT5 determined in this study could contribute to that phenomenon. EAAT5 as a presynaptic glutamate transporter has a large glutamate-gated chloride conductance that could function as a presynaptic feedback inhibitor that improves temporal resolution of synaptic transmission via its glutamate-gated chloride channel function and its impact on presynaptic membrane potential [35,39,41,44,46,85,86,87,88,89,90]. The lack or lower activity of this EAAT5-based feedback mechanism in EAE photoreceptor synapses could contribute to the decreased visual performance in EAE mice (decreased frequence sensitivity) in previously published optometry experiments on MOG/CFA-injected EAE mice [25].

Whether a decreased expression of EAAT5 in EAE photoreceptor synapses as observed in the present study is relevant to prevent a possible spillover of glutamate from the synaptic cleft to extra-synaptic sites remains to be investigated. Recent analyses showed that EAAT5 is a low-capacity glutamate transporter that is rapidly saturated ([44,91,92,93]; for review, [94]). Therefore, glutamate transporters other than EAAT5 could have a stronger impact on a possible spillover of glutamate from synaptic to extrasynaptic sites, particularly at higher rates of vesicular glutamate release at the synapse. It remains to be elucidated whether other glutamate transporters are also decreased at EAE photoreceptor synapses. Recently, it was shown that the GLAST (EAAT1, SLC1A3) glutamate transporter plays an important role in protecting retinal ganglion cells from glutamatergic excitotoxicity in EAE [24]. The GLAST glutamate transporter is a high-capacity glutamate transporter in the retina [95,96] and could also exert an important function to prevent spillover of glutamate from synaptic to extrasynaptic sites at photoreceptor synapses in EAE. This must be investigated by future studies.

### Outlook

In the present study, we showed alterations of the glutamate transporter EAAT5 in the mouse model of multiple sclerosis and demonstrated that the EAAT5 glutamate transporter is less enriched at photoreceptor synapses of MOG/CFA-injected EAE mice in comparison to CFA-injected control mice. EAAT5 is a low-capacity glutamate transporter operating in close vicinity to presynaptic release sites at which exocytosis of glutamatergic synaptic vesicles occurs. EAAT5 is also expressed at the presynaptic terminals of retinal bipolar cells. Retinal bipolar cells are structurally and functionally much more diverse than photoreceptor synapses. Future analyses will show whether the EAAT5 transporter is also compromised at retinal bipolar cell ribbon synapses. The recently generated EAAT5 knockout [46] is an ideal tool to further analyze the functional role of EAAT5 in multiple sclerosis disease development and progression. It will be important to analyze whether other glutamate transporters are affected in EAE, including high-capacity glutamate transporters, that could possibly contribute to photoreceptor synapse pathology. Interestingly, a recent study demonstrated that the GLAST/EAAT1 glutamate is also downregulated in the inner retina in the EAE animal model of MS and that AAV-mediated overexpression of GLAST/EAAT1 protects retinal ganglion cells from cell death [74]. These findings suggest an important role of glutamate transporters for the pathogenesis of multiple sclerosis, and the analyses of glutamate transporters in EAE/MS could help to develop new therapeutic strategies for the treatment of the disease.

## Figures and Tables

**Table 1:** Primary antibodies.

Antibody	Source	References	Dilution
EAAT5 (immunogen affinity-purified rabbit polyclonal) *	Abcam; Cambridge UK; ab230217	n.a.	1:200 (IF) 1:500 (WB)
RIBEYE(B) (mouse monoclonal, clone 2D9)	Lab-made	[25,48]	1:1000 (IF)
Actin (mouse monoclonal antibody, clone C4)	Millipore; Molsheim, France; #1501R	[49]	1:1000 (IF) 1:1000 (WB)
6xHis, HexaHis-tag (mouse monoclonal antibody, clone 1B7G5)	Proteintech;Planegg-Martinsried, Germany; #66005-1-Ig	[50]	1:5000 (WB)

* EAAT5 is a 559 amino acid (aa) long protein in mice (NP_666367.3, GI:1597486091) with a predicted running position at ≈65 kDa in the Western blot (WB) analyses. The affinity-purified rabbit polyclonal EAAT 5 antibody (abcam) was raised against a fusion protein corresponding to amino acid (aa) 100–250 of human EAAT5 (O00341). The specificity of the antibody was verified by a fusion protein that we generated from recombinant synthetic DNA (see below).

**Table 2:** Secondary antibodies.

Antibody	Source	Dilution
Chicken anti-rabbit-Alexa488	Invitrogen, Molecular Probes, Karlsruhe, Germany; A-21441	1:1000 (IF)
Donkey anti-rabbit-Alexa488	Invitrogen, Molecular Probes, Karlsruhe, Germany; A-21206	1:1000 (IF)
Donkey anti-mouse-Alexa568	Invitrogen, Molecular Probes, Karlsruhe, Germany; A-10037	1:1000 (IF)
Goat anti-rabbit-horseradish peroxidase (HRP)	Sigma; Taufkirchen, Germany; A6154	1:5000 (WB)
Goat anti-mouse-horseradish peroxidase (HRP)	Sigma; Taufkirchen, Germany; A3673	1:5000 (WB)

Abbreviations: IF, immunofluorescence microscopy; WB, Western blot.

## Data Availability

All data are presented in the main manuscript and the manuscript figures.
